# The Role of School Leaders’ Health Literacy for the Implementation of Health Promoting Schools

**DOI:** 10.3390/ijerph17061855

**Published:** 2020-03-12

**Authors:** Kevin Dadaczynski, Katharina Rathmann, Thomas Hering, Orkan Okan

**Affiliations:** 1 Department of Nursing and Health Science, Fulda University of Applied Sciences, 36037 Fulda, Germany; katharina.rathmann@pg.hs-fulda.de; 2 Department of Nursing and Health Science, Leuphana University Lueneburg, 21337 Lueneburg, Germany; 3 Department of Applied Human Sciences, Magdeburg-Stendal University of Applied Sciences, 39676 Stendal, Germany; thomas.hering@h2.de; 4 Faculty of Educational Science, Centre for Prevention and Intervention in Childhood and Adolescence, University of Bielefeld, 33615 Bielefeld, Germany; orkan.okan@uni-bielefeld.de

**Keywords:** school leaders, principals, health literacy, health promoting school, implementation

## Abstract

Background: The promotion of health literacy is seen as an urgent goal in public health and education and, hence, should be integrated in the school context as a component of the holistic health promoting school (HPS) approach. However, only limited empirical studies have addressed health literacy of school staff so far. Hence, this study aimed to examine the level of health literacy among school leaders and its association with the extent of HPS implementation. Methods: A cross-sectional study with *n* = 680 school principals and members of the school management board from Germany was carried out at the end of 2018. Individual health literacy, attitudes, and competencies towards HPS and occupational self-efficacy served as independent variables and the level of HPS implementation was the dependent variable. Data were analyzed using univariate and bivariate analysis as well as multiple binary logistic regression. Results: 29.3% of school leaders show a limited health literacy with significantly higher values found for male respondents. Regression analyses revealed that male gender (OR: 1.91, 95% CI: 1.22–2.99), HPS attitudes (OR: 3.17, 95% CI: 2.13–4.72), and HPS competencies (OR: 3.66, 95% CI: 2.43–5.50) were associated with a lower level of HPS implementation. Furthermore, regression analyses differentiated by gender showed that limited health literacy is associated with a low level of HPS implementation for male school leaders only (OR: 2.81, 95% CI: 1.22–6.45). Conclusions: The promotion of health literacy especially for male leaders would not only result in positive effects on an individual level but also could contribute to a stronger implementation of activities on school health promotion. We suggest integrating health literacy, HPS attitudes, and competencies more strongly into the qualification and in further training of school leaders.

## 1. Introduction

Worldwide, schools as learning and teaching environments are recognized as appropriate venues for health promotion and prevention that reach a large proportion of young people, teachers, and nonteaching staff [1]. In addition to isolated, often topic- and target group-specific activities in the school setting, the Health Promoting School (HPS) approach has emerged as a holistic intervention strategy since the adoption of the Ottawa Charta on health promotion [2]. Favored by the World Health Organization (WHO) [3,4] and advocated by the Schools for Health in Europe (SHE) network [5], this approach moves beyond individual behavior by also aiming at whole system changes through, e.g., strengthening the physical and social environment, including interpersonal relationships, school management, policy structures, and teaching and learning conditions. It combines principles of health promotion to address the school structure and all individuals within the school, resulting in a wide range of activities to maintain equity and to mitigate the effects of social and health inequalities [1,6]. In addition, HPS is a key intervention strategy to strengthen health resources and to promote healthy lifestyles in order to reduce risk factors for noncommunicable disease in the life course [3,7]. Although the evidence base is still limited, available research results show that the HPS approach can have positive effects on body composition (e.g., BMI) [8,9,10], healthy eating practices (e.g., fruit and vegetable consumption) [9,10,11,12], physical activity and fitness [9,10,11], or mental health outcomes (e.g., social-emotional competencies and aggressive behavior) [9,11,13].

Compared to highly standardized (i.e., prepackaged) intervention programs, that are delivered with high implementation fidelity, the HPS approach is characterized by context sensitivity that requires adaptations to the specific conditions and dynamic processes of a single school [5,14,15]. Due to the complexity of interventions that are rooted in a broader socio-ecological determination of health, a growing need of a “science of delivery” has been demanded that aims to identify elements and dimensions of high-quality implementation [16]. Amongst a wide array of components, school leaders are increasingly identified as gatekeepers for the initiation and sustainability of standardized and complex interventions on school health promotion. In their evaluation of the Promoting Alternative Thinking Strategies (PATHS) curriculum, Kam et al. [17] reported that high support from school principals was associated with significant higher improvements in pupil’s behaviors. Similarly, another study from the US with *n* = 42 physical education teachers shows that the support of the school principal proved to be a significant predictor for the implementation of the Comprehensive School Physical Activity Program (CSPAP), i.e., the higher the principals’ support, the more components of the CSPAP were implemented [18]. Next to the provision of general support personal knowledge, attitudes and competencies seem to play a crucial role in the implementation. Using data from a multiple-case study, conducted in an Austrian province, Adamowitch et al. [19] reported that school principals were mostly the “driving force” in initiating school health promotion and in deciding (together with the later implementer) for concrete health promoting activities. Personal knowledge and professional experiences had an influence on the decision-making process, i.e., often school principals with active participation in school health promotion had a professional background as physical education, biology, or psychology teacher. Evidence from the Norwegian Network on Health Promoting Schools suggests that a positive attitude of the school leader towards school health promotion was perceived as key for running the program at schools and for sustaining it over time [20,21]. Moreover, current research results from Taiwan with *n* = 1.140 school principals and *n* = 1.110 HPS coordinators (i.e., teachers) indicate that school leaders’ understanding of the HPS and their willingness to sustain HPS at their school were significantly associated with higher levels of HPS implementation and teachers’ willingness to implement HPS on a continuing basis [22]. With regard to prior evidence, it is surprising that there is neither standardized training nor any defined regulations for specific competencies on HPS implementation of school leaders in Germany.

In addition to the abovementioned school leader-oriented influencing factors, the importance of general health-related competencies has been increasingly discussed in public health research in recent years. Today, the research field offers a plethora of health literacy definitions and models, covering a wide array of concepts from narrow functional health literacy through comprehensive, generic health literacy to disease-specific health literacy and topic-related health literacy [23,24]. Despite the variety of definitions, (generic) health literacy can be defined as “people’s knowledge, motivation, and competences to access, understand, appraise, and apply health information in order to make judgments and take decisions in everyday life concerning healthcare, disease prevention, and health promotion.” [23] (p. 3). Epidemiological findings from eight EU countries suggest that almost 47% of the population aged 15 years and older had a limited (inadequate or problematic) health literacy with highest prevalence rates found for Bulgaria (62.1%) and lowest rates for the Netherlands (28.7%) [25]. This and previous studies showed that health literacy follows a social gradient, i.e., people with lower socioeconomic status are more often affected by a limited health literacy [25,26]. With regard to gender differences on health literacy, findings are mixed. While representative German surveys did not report any gender differences [27,28], there are also study findings indicating gender disparities on both functional health literacy [29] and generic health literacy [30]. Results from a national sample of British adults (*n* = 719) revealed that men were more likely to show a limited functional health literacy (OR: 2.04; 95% CI: 1.16–3.5) [29]. Moreover, in their study on generic health literacy with *n* = 585 Korean adults aged 19 years and older, Lee et al. [30] reported that males had more problems in understanding medical forms, directions on medication bottles, and written information offered by health care providers. Regarding health outcomes, research findings demonstrate that limited functional health literacy is associated with harmful and risky health behaviour in adolescence (e.g., nutritional and dieting habits, physical and sedentary activity, substance use, smoking, and alcohol use), whereas higher levels of media health literacy were positively associated with more health promoting behaviours [31]. Research on functional health literacy of adults associated limited levels with more hospitalizations, greater use of emergency care, limited use of early diagnosis and screening, reduced ability to interpret health messages, a poorer overall health status [32], and higher mortality rates in adulthood [33]. In view of this and against the background of its educational links (e.g., educational attainment and academic performance [34]), it is not surprising that the promotion of health literacy is seen as an urgent goal in public health and education and, hence, should be integrated as early as possible in the school context [35,36,37,38].

In contrast to a separate intervention strategy, it is argued that the promotion of health literacy should be seen as a component of the holistic HPS approach and should be developed from within the schools and the educational system [7,38,39]. To achieve this, teachers and nonteaching staff need to be trained to be able to integrate health literacy in the educational core mission and the overall HPS strategy of schools. Teachers’ health literacy is understood to be the counterpart of school children’s health literacy, which is why their health literacy equally needs to be addressed as early as they start preservice training and should continue later on while they are in in-service contexts in order to ensure that they are equipped to deliver quality health literacy teaching in the classroom and to support the development of pupils’ health literacy [40,41]. However, despite the high importance, empirical studies on health literacy of teachers are sparse. Results from a recent cross-sectional study with *n* = 502 secondary school teachers from Sri Lanka revealed a limited health literacy for 31.5% of the respondents with higher percentages found for younger teachers (≤45 years) and those with less teaching experiences (≤10 years) [42]. Moreover, amongst others, higher levels of teachers’ health literacy were associated with being a member of a health club/welfare group or with having done any special course on health. Further findings from Taiwan indicate that teachers’ health literacy teaching beliefs, their attitudes toward health literacy instruction, and their level of confidence in their ability to teach health literacy (but not their individual health literacy level) served as predictors for health literacy teaching intentions [43]. Besides these few quantitative studies, qualitative research also illuminates the critical influence that teachers have on schoolchildren’s health literacy learning in schools. An Australian study showed that, when teachers applied health education techniques and content beyond the transfer of factual health knowledge (e.g., teaching active health literacy skills, providing practical experience shaped to their students’ needs, and delivering health content and information that was relevant to student’s everyday lives), the development of student health literacy was facilitated to a greater degree [41]. Similarly, results from a Canadian study on implementing a health literacy curriculum showed that teachers teaching techniques in relation to health literacy and the quality and quantity of the health information in the classroom mattered most and had influence on pupils’ health literacy learning [44].

However, the few studies available so far relate to the professional group of teachers while no findings are available on the health literacy of school leaders. This is surprising given the fact that this group is of high importance for the entire school and the quality of its teaching and learning conditions, processes, and results [45,46]. Moreover, as indicated above, slowly emerging evidence demonstrates that school principals and managers are gatekeepers for the initiation and sustainable anchoring of activities on school health promotion [47].

Against this background, the present study aims to explore the health literacy of school leaders and its association with the level of HPS implementation. In most studies mentioned above, school leaders have been regarded as a homogeneous group, i.e., subgroup differences within this occupational group are rarely employed. Since primary schools are more oriented towards the holistic development of the child, it would be reasonable to assume that primary school leaders support activities on school health promotion more strongly, resulting in higher HPS implementation. In this context, the results of a German study showed that primary school leaders indicated the highest and that secondary school leaders indicated the lowest need for support in the area of school health promotion [48]. Moreover, it can be assumed that the professional role of school leaders is associated with the implementation status of HPS. Since school principals are required to teach to a lesser extent than other members of the school leadership team (e.g., deputy school principals), they may be able to devote more time resources to supporting school health promotion. However, as available empirical studies on health literacy and HPS implementation have not differentiated between different leadership positions and failed to consider differences between the type of school, both aspects should be considered as covariate.

Specifically, the following research questions are addressed:

1. What is the level of health literacy among school leaders?
2. What sociodemographic and work-related characteristics are associated with health literacy of school leaders?
3. What is the relationship between health literacy of school leaders and the level of HPS implementation taking into account sociodemographic characteristics and other school leader-oriented factors (HPS related attitudes, competencies, and self-efficacy)?

## 2. Materials and Methods

### 2.1. Study Population, Data Collection, and Study Design

This cross-sectional study was part of a larger survey which aimed to investigate the work and health situation of German school leaders and to examine their role in the implementation of school health promotion. The study was carried out for the first time as an online survey in the German federal state North Rhine-Westphalia from late 2012 to early 2013. Due to the high response rate (*n* = 2.039), the study was also implemented in the federal states of Berlin (*n* = 237), Lower Saxony (*n* = 1.336), and Schleswig-Holstein (*n* = 714) from 2014 to 2016 in cooperation with the respective school leadership associations. To ensure comparability, a core questionnaire was used in each study, which was supplemented by additional scales and items.

This paper refers to the data of the latest study conducted in the federal state of Hesse from October to December 2018, which for the first time examined the health literacy of school leaders. The target group was school principals and members of the school management boards (e.g., vice-principals) in general and vocational schools. The study protocol was approved by the Hesse Ministry of Education. All eligible participants were invited by email and newsletter, including two reminders by the Hesse association of school principals. The survey was administered electronically using the Enterprise Feedback Suite (EFS) survey tool by Questback. Participation was voluntary, and anonymity was assured. Upon entering the survey site, participants were presented with information regarding the aims and the background of the study. After checking a consent box at the bottom of the page, participants were directed to the questionnaire.

### 2.2. Measures

General **demographic and work characteristics** include gender (male, female), age (≤45 years, 46 to 60 years, and >60 years), type of school (primary school, grammar/high school (gymnasium), schools for children with special educational needs (e.g., disabilities), vocational school, others (e.g., comprehensive and mixed schools), and professional role (school principal and member of the school management board).

**Health literacy** as an independent variable was assessed using the German short version of the European Health Literacy Survey Questionnaire (HLS-EU-16) [49,50]. The 16 items were based on Rasch modelling, and item selection was based on content validity. They refer to various tasks and activities related to health care, disease prevention, or health promotion, with respondents assessing how simple they think the task or activity is (1 = very simple and 4 = very difficult). One example item is “On a scale from very easy to very difficult, how easy would you say it is to find information on how to manage mental health problems like stress or depression?” Compared to the long version with 47 items, the short version does not allow to calculate health literacy scores for each subdimension but shows (as an overall scale) a high concurrent validity with the long version (*r* = 0.82) [49,50]. The internal consistency (Cronbach’s α) for the overall scale was 0.90. According to the scale developer, all items were dichotomized (1 = very simple/ fairly simple and 0 = fairly difficult/ difficult) and then summarized as a sum score ranging from 0 to 16 points [49,50]. No sum score was calculated for respondents with more than two missing values. Based on the sum score, three levels of health literacy are suggested: inadequate health literacy (0 to 8 points), problematic health literacy (9 to 12 points), and sufficient health literacy (13 to 16 points). These cut-off values are adapted from the four health literacy levels of the long version and have been used in a number of studies [28,51]. For all analyses, inadequate and problematic health literacy were combined to the category limited health literacy.

Based on the existing evidence on the association of school principals on HPS implementation, we included attitudes, competencies, and self-efficacy as independent variables. Adapted from a study on workplace health promotion, **personal attitudes towards HPS** were measured with six items [52]. These included the perceived importance of the respondents to promote the health of pupils and teachers or the perceived importance of health for the work and learning ability, e.g., “I think it is important that the working and learning conditions at my school are created in a health promoting way for the school staff and the pupils.” All items could be assessed on a 5-point Likert scale ranging from 1 (completely disagree) to 5 (completely agree). Cronbach’s α for this variable was 0.76. **HPS related competencies** were operationalized with three items adapted from Wilde et al. [52] that captured the perceived ability to promote the health of pupils and the school staff. An example item was “I am not sure how I can support activities on school health promotion and prevention at my school.” Due to the negative formulation, all items were inverted, with higher values (1 = completely disagree and 5 = completely agree) indicating a higher level of HPS competence. The internal consistency (Cronbach’s α) for the HPS competencies scale was 0.82. Finally, to capture subjective beliefs to successfully fulfil a certain task or to cope with demands and problems, we used the **occupational self-efficacy** scale by Abele et al. [53]. In contrast to general self-efficacy, the scale is domain specific and refers to the subjective abilities to successfully fulfil job-related tasks. It comprises six items that could be rated on a five-level response scale ranging from 1 (not true) to 5 (completely true) with higher values indicating higher levels of occupational self-efficacy, e.g., “I know exactly that I can meet the demands placed on my profession if I only want to.” The psychometric quality of this scale was successfully tested in previous studies [53], and in this study, an acceptable Cronbach’s α of 0.73 was achieved. For further regression analyses, all three indicators were dichotomized using median-split (1 = low level of attitudes/competencies/self-efficacy and 0 = high level of attitudes/competencies/self-efficacy).

The implementation level of the HPS served as dependent variable in this study. **Level of HPS implementation** was operationalized using an 8-item scale by Harazd et al. [54], which could be answered on a 4-point Likert-type scale (1 = strongly disagree and 4 = strongly agree). In order to capture different facets of the holistic HPS approach, the existing scale was extended by 6 self-formulated items. After developing additional items within the research group, the extended scale was pretested with selected school principals and teachers (*n* = 6). Based on their feedback, slight changes in the formulations were made. Two example items are “Health promotion and health goals are integrated in the mission statement and program of our school.” and “At our school, pupils are supported in the development of health-promoting behaviour.” The internal consistency (Cronbach’s α) for the overall scale was 0.90. For further regression analyses, this indicator has been dichotomized using median-split (1 = low level of HPS implementation and 0 = high level of HPS implementation).

### 2.3. Statistical Analyses

All statistical analyses were performed using IBM SPSS version 23 (IBM, New York, NY, USA). First, we analysed the data descriptively (i.e., means, standard deviations, and frequencies). Second, all potential sociodemographic and work characteristics were cross tabulated with the two levels of health literacy (limited vs. sufficient) and their associations were assessed using chi square tests (*χ*^2^). Moreover, Pearson correlation (two-tailed) for all independent and dependent variables were conducted. Finally, multiple binary logistic regression analyses were used to examine the association of all explanatory variables (sociodemographic and work characteristics, HPS attitudes, competencies, self-efficacy, and health literacy) with the level of HPS implementation by odds ratio (OR) and its respective 95% confidence interval (95% CI). This form of regression analysis was chosen because there are no predefined cut-off values for the dependent variable resulting in an empirical (artificial) division of low versus high HPS implementation (using median-split). All independent variables were included block-wise. Block 1 covered sociodemographic and work characteristics, while block 2 (in addition to the variables of block 1) included HPS attitudes, competencies, and self-efficacy. Block 3 included health literacy in addition to the variables of the previous blocks. The estimated fit of the regression models was provided by Nagelkerke’s R squared. Again, chi square tests were used for testing the significance of the different models. For all analyses *p*-values < 0.05 were considered statistically significant.

## 3. Results

### 3.1. Demographic and Work Characteristics

A total of *n* = 680 respondents completed the survey. The majority were female (67.2%), between the age of 46 to 60 years (60.7%), and had the role of the main school principal (81.2%). Moreover, almost half of respondents were from primary schools (48.5%), another 10% worked at a grammar school, and about 9% worked at a school for children with special educational needs (see Table 1 for participants’ demographics).

### 3.2. Results of Uni- and Bivariate Analyses

Based on the calculation of the health literacy levels, more than two thirds of all respondents (70.7%) reported a sufficient level of health literacy, while 23.5% had a problematic and 5.8% an inadequate health literacy. Table 2 shows health literacy levels (i.e., problematic and inadequate health literacy were combined to limited health literacy), stratified by sociodemographic and work characteristics.

With regard to gender, chi square tests revealed a significant difference with a higher percentage of limited health literacy found for male compared to female leaders (34.9% vs. 26.5%, *χ*^2^(1) = 4.87, *p* < 0.05). While no differences in health literacy could be identified between school principals and members of the management board (e.g., vice-principals), descriptive results showed a higher percentage of having a limited health literacy for middle aged respondents (45 to 60 years, 31.7%) and respondents from vocational schools (31.4%). However, differences among age groups and type of school did not prove to be significant.

A summary of the means (*M*), standard deviations (*SD*), and Pearson correlations of all explanatory variables and the dependent variable is shown in Table 3. There were small but statistically significant associations between health literacy and level of HPS implementation (*r* = 0.11, *p* < 0.001), HPS competencies (*r* = 0.12, *p* < 0.001), and occupational self-efficacy (*r* = 0.16, *p* < 0.001). Furthermore, level of HPS implementation as dependent variable correlated on a moderate level with HPS attitudes (*r* = 0.34, *p* < 0.001) and HPS competencies (*r* = 0.41, *p* < 0.001), while small associations with occupational self-efficacy (*r* = 0.12, *p* < 0.001) could be found.

### 3.3. Results of Multivariate Analyses

All variables from bivariate (correlation) analyses with significant associations (*p* < 0.05; see Table 3) were included in the multiple binary regression model with level of HPS implementation as the dependent variable (low level of HPS implementation = 1 and high level of HPS implementation = 0). With regard to sociodemographic and work characteristics (block 1), only gender and professional role were significantly associated with the implementation status of the HPS (see Table 4). Compared to female respondents, we observed a 1.91-fold increased chance (“risk”) of low HPS implementation for male school leaders (95% CI: 1.22–2.99). Moreover, not belonging to the group of school principals (member of the school management team, e.g., vice-principals) was associated with a more than 3.5-fold increase in the probability of a low level of HPS implementation (OR: 3.63, 95% CI: 1.95–6.76). For block 2, attitudes and competencies showed significant associations with the implementation status, i.e., respondents with low HPS attitudes and low HPS competencies also reported a lower HPS implementation for their schools (HPS attitudes = OR: 3.17, 95% CI: 2.13–4.72; HPS competencies = OR: 3.66, 95% CI: 2.43–5.50). Finally, added in block 3, we found significant associations between the level of health literacy and the implementation status of the HPS. Compared to respondents with sufficient health literacy, school leaders with limited health literacy had a 1.61-fold increased risk of a low HPS implementation (95% CI: 1.04–2.49). Nagelkerke’s R^2^ showed a value of 0.30, indicating that about one third of the variation between the two groups (low and high HPS implementation status) was explained by the explanatory variables of the full model (block 3).

Based on the identified gender differences in the level of health literacy and the gender-related associations with the HPS implementation status (Table 3 and Table 4), we calculated multiple binary logistic regressions stratified by gender. Subsequent significance tests of the difference between significant regression coefficients in women and men were tested two-tailed using the z-two-proportion-test. As shown in Table 5, membership of the school management board (e.g., vice principal) was significantly associated with lower HPS implementation for female and male respondents (male = OR: 4.43, 95% CI: 1.47–13.35; female = OR: 3.81, 95% CI: 1.74–8.34).

For block 2, a heterogeneous picture emerged for male and female respondents. Although HPS attitudes and competencies were significantly linked with the dependent variable for both genders, the size of the odds ratios differed in the gender comparison. While male school leaders with low HPS attitudes showed a higher increase of the probability of a low level of HPS implementation (male = OR: 4.24, 95% CI: 1.92–9.28; female = OR: 3.07, 95% CI: 1.90–4.94), the opposite could be observed with regard to HPS competencies with higher odds ratios found for female leaders (male = OR: 2.81, 95% CI: 1.27–6.23; female = OR: 4.21, 95% CI: 12.59–6.86). However, two-tailed difference tests of the odds ratios revealed no significant gender differences (HPS attitudes: *z* = 1.57, *p* > 0.05; HPS competencies: *z* = −1.82, *p* > 0.05). When health literacy was entered in block 3, significant associations between limited health literacy and low-level HPS implementation could only observed for male school leaders (OR: 2.81, 95% CI: 1.22–6.45). Nagelkerke’s R^2^ of 0.30 (male) and 0.29 (female) indicate that about one third of the variation between the two groups (low and high HPS implementation status) was explained by the explanatory variables of the full model (block 3).

## 4. Discussion

The present study aimed to examine, firstly, the level of health literacy of school leaders and its connection with the implementation status of health promotion in schools. Our results revealed that 29.3% of the school principals and administrators had a limited level of health literacy. Compared with the results of representative German surveys, the percentage of respondents with limited health literacy is substantially lower. For example, 54.3% of the respondents (*n* = 2.000) in the German Health Literacy Survey (HLS-GER) had a limited health literacy [27], while the percentage for this subgroup was 44.2% in the German Health Update (GEDA) study (*n* = 4.854) [28]. Differentiated by educational level, the GEDA study showed that 38.2% of those with a high educational status had a limited health literacy (compared to 48.4% of respondents with low level of education). Although the proportion of respondents (as a group with high educational status) with limited health literacy is lower in our study, the findings suggest a clear public health need to promote individual health literacy of this occupational group. This is not least due to the fact that school leaders act as role models within their schools. Next to findings suggesting an important role of leadership on employee health and satisfaction [55], research results from the educational field found that wellbeing of school principals is significantly associated with the wellbeing of teachers (particularly in primary schools) [54]. Against the background of the link between health literacy and health outcomes, it can be assumed that strengthening health literacy not only is beneficial to the health of school leaders but also can have positive effects on the health of the entire school staff through health-oriented leadership behaviour [56].

Secondly, there is a particular need for support especially for male school leaders, who are more often affected by limited health literacy than female respondents (36.5% vs. 26.9%). It is argued that these gender differences can be traced back to the traditional division of gender roles, which ascribes greater responsibility to women for the health of family members (e.g., child and elderly care) [30,57,58]. In addition, women make more frequent use of health promotion, prevention, and health care services compared to men, which could lead to women having greater health knowledge and greater routine in using the health care system [59,60].

Third, besides initial findings on the level of health literacy, the main results of our study indicate that health literacy is linked to the implementation status of the HPS even after controlling for sociodemographic and work characteristics as well as further school-leader oriented influencing factors. School leaders with sufficient health literacy levels were more likely to report a higher level of HPS implementation. They may be able to apply their individual-level abilities (to find, understand, critically reflect, and apply health-related information) to an organisational context to support a variety of activities on school health promotion. Thus, our findings somewhat contradict the results of the study by Lai et al. [43], in which health literacy was not associated with the intention of teachers, to implement health literacy instruction within the following year. However, it should be noted that the health literacy measurement and its underpinning model as used by Lai et al. was based on a different concept of health literacy than in our study. The findings of our study are remarkable in that we did not follow a focused instructional approach but rather pursued a holistic approach on school health promotion involving different target groups (pupils and teachers) and activities as dependent variable. Moreover, in our separated regression analyses, we found gender differences in health literacy and an association between gender and HPS implementation. Interestingly, a limited health literacy was linked with a low implementation status of HPS for male school leaders only. This finding is important in two respects: in comparison to female school leaders, male respondents are more often affected by limited health literacy and a limited health literacy appears to hinder support for health-promoting activities among male school leaders. Hence, the promotion of health literacy especially for males not only would result in positive effects on an individual level but also could contribute to a stronger implementation of activities on school health promotion.

With regard to the school-leader-oriented influencing factors, our results confirm the findings of previous studies [19,20,21,22]. HPS attitudes and competencies served as significant predictors for HPS implementation for both male and female school leaders. While health literacy tends to encompass the cognitive skills needed for self-directed search for and dealing with health-related information, including the use of such information to make healthy choices [23], HPS competencies focus more on action competencies, i.e., concrete capacities that allow people to perform certain actions (e.g., on school health promotion) [61]. Although both constructs are related to each other (see Table 3), they need to be regarded as distinct concepts, each of which is independently associated with the implementation status of HPS. In previous research, various efforts have been made to develop core competencies in health education and school health promotion for teachers. Using the Delphi method, Moynihan et al. [62] suggested 12 core competencies for health education teachers in supporting the development of health literacy among their pupils. These have been grouped into three categories and include knowledge (e.g., knowledge of health education/promotion theories and models or knowledge of health education curricula), skills (e.g., skills in planning, implementing and assessing whole school health promoting initiatives), and attitudes (e.g., willingness to engage in whole school and community health promoting activities). Regardless of these efforts, the competence grids described above refer to teachers, while for school leaders, no core competencies on health education and HPS have been presented so far. Currently, there is no comprehensive and standardized training for the work as a school principal in Germany [63]. Teachers undergo one-off further training before taking up their leadership position, but the nature of this varies substantially between the German federal states. For the federal state of Hesse, the qualification phase for prospective school principals comprises five modules (1. communication, 2. effective curriculum-based leadership, 3. management of the school budget, 4. school legislation, and 5. management of change processes) with a duration of 2 to 3 days each, which are completed over the course of approximately 12 months. The focus is on the promotion of professional competence and the strengthening of personal and communicative skills with references to health especially in the area of personal and professional competencies (e.g., promotion of individual resilience and strengthening of salutogenic action competence). However, health as a cross-cutting issue has not yet been sufficiently addressed in the qualification of school principals.

When interpreting the results of our study, several limitations need to be considered. First, the sample is not representative and the results cannot be generalised to the whole population of school leaders in the federal state of Hesse. According to the school statistics for the 2017/18 school year, there were 1804 schools in Hessen, of which more than half (57%) are primary schools. A further 10% of all schools from Hesse was schools for children with special educational needs, while 7% was grammar/high schools and 6% was vocational schools. Due to missing public statistics, no comparisons can be made between the study sample and the general population of school principals and administrators from the federal state of Hesse. Moreover, it should also be taken into account that, due to the group of eligible participants addressed (school principals and members of the school management), more than one person per school were able to participate in the survey. This could have led to a positive bias, as, e.g., leaders from well-implemented HPS felt more strongly addressed and motivated to participate. Second, based on the cross-sectional design of this study, the results concerning the association between health literacy and level of HPS implementation cannot be interpreted as causal relationships. This is of particular importance as there is no empirical clarity about the multifaceted relationship between health literacy and health promotion so far [64]. Besides the assumption that a high level of health literacy has a positive effect on health promotion activities in schools, it is also conceivable that a higher level of HPS implementation could be beneficial for the health literacy of school leaders. Finally, school leaders were the only source of information in this study, which may have led to limitations in validity. For example, studies indicate that health promotion in schools is often implemented by individual, particularly committed teachers, and school principals as gatekeepers are only marginally involved [19]. Hence, consideration of an additional external source (e.g., teachers) could have contributed to a more validated assessment of the state of HPS implementation.

## 5. Conclusions

This study adds to the existing research in various ways. To our knowledge, this is the first study examining the health literacy of school principals and school managers. In their overall responsibility for the entire school, school principals serve as gatekeepers for any school (health) development process and, hence, need to be put more firmly in the focus of school health promotion research. Although school leaders belong to a group with a high level of education, our results indicate that slightly less than a third of the respondents are affected by a limited health literacy and, hence, should be supported in strengthening their individual abilities to search for and to deal with health-related information, including their use and healthy decision making. On the school level, activities should focus on implementing a culture for health literacy embedded within the HPS approach, whereas on the individual level, activities should focus specifically on male school leaders, who more often have a limited health literacy compared to female respondents. Moreover, it could be shown for the first time that individual health literacy is associated with holistic practices on school health promotion, thus placing individual health literacy more in the context of a socio-ecological understanding of health. Gender differences found in this study underline the need for gender-specific actions to strengthen individual health literacy. Next to the promotion of health literacy, these activities should also encompass strategies to strengthen attitudes and competencies towards HPS. These could be achieved by integrating these issues more strongly into the qualification phase and in further training of school leaders. Finally, further research is needed to confirm the study results presented here (e.g., with regard to gender disparities) and to examine causal relationship between health literacy and organisational practices on school health promotion.

## Figures and Tables

**Table 1:** Characteristics of study participants.

Item	Category	Frequency (n)	Percentage (%)
Gender	Male	223	32.8
	Female	457	67.2
Age	≤45 years	146	21.5
	46 to 60 years	413	60.7
	>60 years	121	17.8
Professional Role	School principal	552	81.2
	Member of management board	128	18.8
Type of School	Primary school	330	48.5
	Grammar/high school (Gymnasium)	65	9.6
	Schools for children with special educational needs	57	8.4
	Vocational school	54	7.9
	Other	174	25.6

**Table 2:** Health literacy levels of school principals stratified by sociodemographic and work characteristics.

	Limited HL	Sufficient HL		
	% (n)	% (n)	χ^2^ (df)	*p*
***Gender* (*n* = 656)**			4.871 (1)	<0.05
Male	34.9 (75)	65.1 (140)		
Female	26.5 (117)	73.5 (324)		
***Age* (*n* = 656)**			2.965 (2)	n.s
≤45 years	26.3 (36)	73.7 (101)		
46 to 60 years	31.7 (127)	68.3 (274)		
>60 years	24.6 (29)	75.4 (89)		
***Professional role* (*n* = 656)**			0.017 (1)	n.s.
School principal	29.2 (156)	70.8 (379)		
Member of the manag. board	29.8 (36)	70.2 (85)		
***Type of school* (*n* = 656)**			0.565 (4)	n.s
Primary school	29.6 (94)	70.4 (224)		
Gramar/high school (Gymnasium)	25.8 (16)	74.2 (46)		
Schools for children with special educational needs	27.8 (15)	72.2 (39)		
Vocational school	31.4 (16)	68.6 (35)		
Others	29.8 (51)	70.2 (120)		
**Total (*n* = 656)**	**29.3 (192)**	**70.7 (464)**		

**Notes.** HL: health literacy, *χ*^2^: Chi Square, df: degrees of freedom, n.s.: not significant, %: percent, n: frequency.

**Table 3:** Means (*M*), Standard Deviations (*SD*), and correlations of dependent and independent variables.

	*M*	*SD*	1	2	3	4	5
1. Level of HPS implementation	2.71	0.56	(0.90)				
2. HPS attitudes	4.48	0.44	0.34 **	(0.76)			
3. HPS competencies	2.88	0.91	0.41 **	0.01	(0.82)		
4. Occupational self-efficacy	3.81	0.63	0.12 **	0.06	0.27 **	(0.73)	
5. Health literacy ^1^	13.50	2.75	0.11 **	0.00	0.12 **	0.16 **	(0.90)

**Notes.** n = 635–677, ^1^ mean values for health literacy are based on the sum score (range: 0–16) internal consistency (α) estimates are on the diagonal, ** *p* < 0.01 * *p* < 0.05; two-tailed tests.

**Table 4:** Multiple binary logistic regression analysis for level of HPS implementation.

	Low Level of HPS Implementation		
	OR	95% CI	R^2^ X^2^ (df) p
***Gender* (*n* = 656)**			
Female	1.00	-	**Block 1** 0.14 60.33 (8) < 0.01 **
Male	1.91 **	**1.22–2.99**	
***Age* (*n* = 656)**			
>60 years	1.00	-	
46 to 60 years	0.89	0.53–1.49	
≤45 years	0.78	0.41–1.49	
***Professional role* (*n* = 656)**			
School principal	1.00	-	
Member of the management board	3.63 **	**1.95–6.76**	
***Type of school* (*n* = 656)**			
Primary school	1.00		
Gramar/high school (Gymnasium)	1.01	0.46–2.22	
Schools for children with special educational needs	0.40	0.18–0.88	
Vocational school	0.90	0.41–1.97	
Others	0.94	0.57–1.55	
**HPS Attitudes**			**Block 2** 0.29 136.05 (11) <0.01 **
High	1.00		
Low	3.17 **	**2.13–4.72**	
**HPS Competencies**			
High	1.00		
Low	3.66 **	**2.43–5.50**	
**Occupational Self-efficacy**			
High	1.00		
Low	1.18	0.79–1.77	
**Health Literacy**			**Block 3** 0.30 140.66 (12) < 0.01 **
Sufficient	1.00		
Limited	1.61 *	**1.04–2.49**	

**Notes.** block 1: gender, age, professional role, and type of school; block 2: block 1 + HPS attitudes, competencies, and self-efficacy; block 3: block 1, 2 + health literacy; HPS: Health Promoting School, OR: Odds Ratios, CI: confidence interval, ** *p* < 0.01, * *p* < 0.05.

**Table 5:** Multiple binary logistic regression analysis for level of HPS implementation stratified by gender.

	Low Level of HPS Implementation				
	Male		Female		
	OR	95% CI	OR	95% CI	*z*, *p*
***Age* (*n* = 656)**					
>60 years	1.00		1.00		
46 to 60 years	0.58	0.27–1.47	1.11	0.58–2.10	
≤45 years	0.51	0.15–1.71	0.91	0.41–2.01	
***Professional Role* (*n* = 656)**					*z* = 0.82, >0.05 (n.s.)
School principal	1.00		1.00		
Member of the management board	4.43 **	**1.47–13.35**	3.81 **	**1.74–8.34**	
***Type of School* (*n* = 656)**					
Primary school	1.00		1.00		
Gramar/high school (Gymnasium)	0.67	0.19–2.39	1.43	0.51–4.00	
Schools for children with special educational needs	0.43	0.11–1.65	0.32 *	**0.11–0.93**	
Vocational school	0.61	0.17–1.65	1.17	0.39–3.48	
Others	1.20	0.46–3.14	0.78	0.42–1.45	
**HPS Attitudes**					*z* = 1.57, >0.05 (n.s.)
High	1.00		1.00		
Low	4.24 **	**1.92–9.28**	3.07 **	**1.90–4.94**	
**HPS Competencies**					*z* = −1.82, >0.05 (n.s.)
High	1.00		1.00		
Low	2.81 *	**1.27–6.23**	4.21 **	**2.59–6.86**	
**Occupational Self-efficacy**					
High	1.00		1.00		
Low	1.77	0.80–3.94	1.07	0.66–1.74	
**Health Literacy**					
Sufficient	1.00		1.00		
Limited	2.81 *	**1.22–6.45**	1.31	0.77–2.23	

**Notes**. Male (*n* = 172), Female (*n* = 376), block 1: gender, age, professional role, and type of school (Nagelkerke’s R^2^ = male: 0.09, female: 0.12); block 2: block 1 + HPS attitudes, competencies, and self-efficacy (Nagelkerke’s R^2^ = male: 0.26, female: 0.29); block 3: block 1, 2 + health literacy (Nagelkerke’s R^2^ = male: 0.30, female: 0.29); HPS: Health Promoting School, OR: Odds Ratios, CI: confidence interval, n.s.: not significant, *z*-score: normal distributed z statistic, ** *p* < 0.01, * *p* < 0.05.
